# Polyostotic Fibrous Dysplasia with Epiphyseal Involvement in Long Bones: A Case Report

**DOI:** 10.1155/2013/715402

**Published:** 2013-04-08

**Authors:** Tomoaki Fukui, Teruya Kawamoto, Toshiaki Hitora, Yoshiki Yamagami, Toshihiro Akisue, Tetsuji Yamamoto

**Affiliations:** ^1^Department of Orthopaedic Surgery, Kagawa University, 1750-1 Ikenobe, Miki-cho, Kida-gun, Kagawa 761-0793, Japan; ^2^Department of Orthopaedic Surgery, Kobe University Graduate School of Medicine, 7-5-2 Kusunoki-cho, Chuo-ku, Kobe Hyogo 650-0017, Japan

## Abstract

Fibrous dysplasia (FD) is an uncommon, but well-known benign skeletal disorder. In cases affecting long bones, FD is commonly recognized to locate in the diaphyses or the metaphyses and to spare the epiphyses. In this paper, we present a rare case of polyostotic FD in a 13-year-old girl with unilateral multiple epiphyseal lesions arising in the femur, the tibia, and the fibula with the growth plates.

## 1. Introduction

Fibrous dysplasia (FD) is a sporadic, tumor-like, benign skeletal disorder originally described by Lichtenstein in 1938 [1] and by Lichtenstein and Jaffe in 1942 [2]. FD is often asymptomatic. However, FD often leads to bone pain, deformities, or pathological fractures. FD is classified into two types: monostotic FD affects only a single bone, and polyostotic FD presents the involvement of multiple bones. While small monostotic FD lesions occur more frequently than polyostotic FD, they result in fewer complications. Conversely, polyostotic lesions tend to enlarge and result in severe complications [3, 4]. In cases affecting tubular bones, FD is commonly recognized to occur in the diaphyses or the metaphyses and to spare the epiphyses [5, 6]. In the present study, we describe a rare case of polyostotic FD with unilateral multiple epiphyseal lesions arising in long bones with the growth plates.

## 2. Case Presentation

A 13-year-old girl was referred to our department with dull pain in the right knee while in motion, which she had noticed approximately one month before her initial visit. There was no pain at rest, swelling, or tenderness in the lower right limb. The range of motion was not limited in the right hip, the right knee, or the right ankle. There was no history of endocrine dysfunction including precocious puberty, and no cutaneous abnormality was found. 

**Figure 1:**
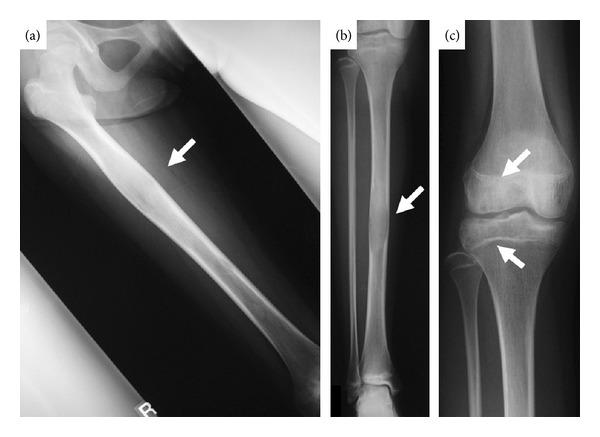
Plain radiographs of the right leg. Radiolucent lesions with sclerotic contours in the diaphyses of the right femur (a) and the right tibia (b) and the epiphyses of the right knee (c).

**Figure 2:**
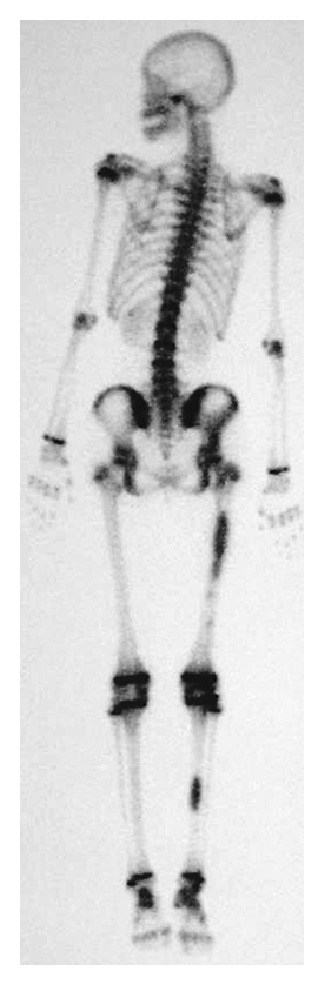
Technetium bone scintigram. Abnormal uptakes are shown in the diaphyses of the right femur and the right tibia and around the bilateral knees and ankles.

**Figure 3:**
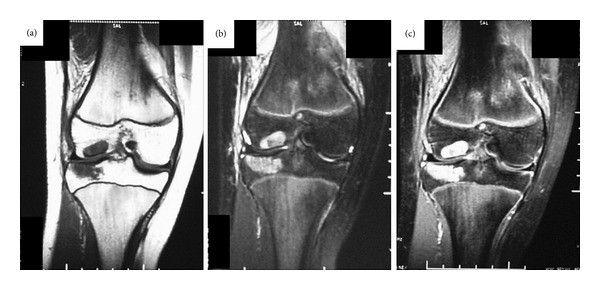
Magnetic resonance imaging (MRI) of the lesions in the distal epiphysis of the right femur and the proximal epiphysis of the right tibia. All lesions show a low signal intensity on a T1-weighted image (a) and a high signal intensity on a fat-suppressed T2-weighted image (b). The lesions were strongly enhanced by gadolinium ((c) T1-weighted images enhanced by gadolinium).

Radiographs of the right lower limb revealed multiple radiolucent lesions with sclerotic contours in the diaphyses of the femur the tibia, the patella, the pelvis, and the tarsal bones. Characteristic lesions were also found in the distal epiphysis of the femur, and the proximal and distal epiphyses of the tibia. The growth plates in both the femora and the tibia had not been closed. The lesions in the diaphyses of the right femur and the right tibia were distended causing the surrounding cortex to thin (Figure 1). Technetium bone scintigram showed increased uptake in the diaphyses of the right femur and the right tibia and the right pelvis and around the bilateral knees and ankles (Figure 2). On magnetic resonance imaging (MRI), the epiphyseal lesions of the distal femur and the proximal tibia showed a low signal intensity on T1-weighted images and a high signal intensity on fat-suppressed T2-weighted images, and the lesions were markedly enhanced by gadolinium (Figure 3). 

**Figure 4:**
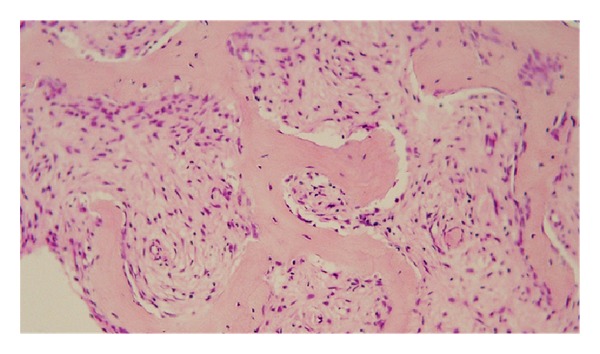
Microscopic findings of the excised lesion in the distal epiphysis of the right femur. Spindle cells grow in storiform pattern around haphazard bony trabeculae and fibrous tissue stroma. There is lack of osteoblast rimming surrounding the trabeculae. (hematoxylin and eosin staining; ×400).

**Figure 5:**
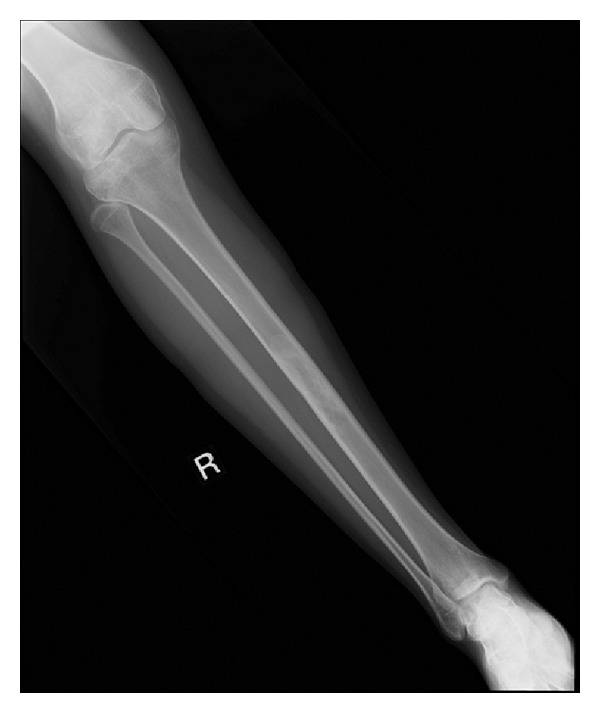
Plain radiographs of the right knee five years after the first surgery.

On the basis of radiological findings, benign bone tumors, such as polyostotic FD or nonossifying fibroma, were suspected. The patient underwent curettage and filling with granules of beta-tricalcium phosphate (*β*-TCP) for the lesion in the diaphysis of the right femur, and the histological diagnosis was FD. Two months after the first surgery, surgeries for the lesions in the diaphysis of the right tibia and in the epiphysis of the right femur were performed. Curettage and filling with granules of *β*-TCP were performed for the lesion of the tibia, which was diagnosed as FD by postoperative histological examination. As FD does not usually arise in the epiphysis, an open biopsy alone was performed for the distal epiphyseal lesion of the right femur in order to make a definitive diagnosis. The lesion in the epiphysis of the distal femur was histologically composed of haphazard bony trabeculae and fibrous tissue stroma containing benign spindle cells. The trabeculae consisted of immature woven bone, the form of which resembled “alphabet soup.” There was lack of osteoblast rimming surrounding the trabeculae (Figure 4). A histological diagnosis of FD was made. Three months after the second surgery, the patient fell and sustained a fracture in the proximal diaphysis of the right femur and underwent osteosynthesis using an intramedullary nail. There was no recurrence in the lesions in the diaphyses of the right femur and the right tibia, and the epiphyseal lesions of the distal femur and the proximal tibia showed no increase in size radiographically at the final follow-up examination (Figure 5).

## 3. Discussion

FD is an uncommon, but well-known benign skeletal disorder, which is considered to be a pathological condition as a result of developmental failure in the remodeling of immature bone to mature lamellar bone and of inappropriate bone realignment in response to a mechanical stress. The disorder of bone maturation leaves a mass of immature isolated trabeculae (woven bone) in dysplastic fibrous tissue [7]. The true incidence of FD is unclear. However, it has been reported that FD represents approximately 5 to 7% of benign bone tumors [3]. FD can occur as monostotic or polyostotic lesions. The latter form of FD is rarely accompanied by endocrine dysfunction, including precocious puberty and cafe-au-lait spots such as characteristic cutaneous abnormalities, known as McCune-Albright syndrome. According to previous reports, the proportion of monostotic FD, polyostotic FD, and McCune-Albright syndrome is approximately 50%–70%, 20%–30% and 3%–10%, respectively [3, 4, 8–13]. As a rule, both monostotic and polyostotic lesions affecting long bones such as the tibia, the femur, or the humerus occur in the diaphyses or the metaphyses. Albright first postulated that the FD bone lesions would spare the epiphyses [14], and several subsequent authors have also reported that epiphyses are not usually affected by FD [5, 6, 15]. On the other hand, several authors have reported that lesions in diaphyses or metaphyses can progress with growth and result in the involvement of the epiphyseal area in adults [5, 6, 15–17]. However, cases of FD with epiphyseal involvement before puberty are quite rare; we found only 5 cases in previous reports [14, 16, 18, 19] (Table 1). Epiphyseal involvement in the proximal tibia was shown in 3 of 5 cases, and the current case is the only case that presents epiphyseal lesions in both the distal femur and the distal tibia. Harris et al. reported two cases of polyostotic FD with epiphyseal involvement. However, he did not describe the pathological findings of the involvement [16]. Nixon and Condon also presented two cases of polyostotic FD with epiphyseal lesions and reported that fibroosseous aberration initially occurred in the epiphyseal growth plate and occasionally showed a bidirectional extension into the metaphysis and epiphysis across the growth plate [18]. In the current case, all the long bones with epiphyseal involvements had diaphyseal or epiphyseal lesions, which is compatible with the theory of Nixon and Condon concerning the advancement of FD. 

FD lesions are structurally fragile and often present in pathological fractures and in cases involving deformities. FD in the epiphyseal area is likely to induce roughened articular surface. Therefore, it is necessary to observe symptoms and radiographic findings carefully. In the current case, an open biopsy was performed for the distal epiphyseal lesion in the right femur, and the lesions in the proximal and the distal epiphyses of the right tibia were managed by observation only. Although the course was not problematic, we should consider that these lesions may affect articular surface and may require long-term observation.

Although the case presented in the current study is extremely rare, we should take notice of two points in order to prevent oversight: one is to include FD in differential diagnoses when polyostotic tumors or tumor-like lesions occur in epiphyses. The other is to verify whether there is epiphyseal involvement or not when suspecting FD. 

## Figures and Tables

**Table 1:** Summary of 5 cases of FD with epiphyseal lesions and the current case.

Case	Sex	Initial complaint	Age of onset	Age at followup	Pigmentation	Precocious sexuality	Location of epiphyseal involvement	Monostotic or polyostotic	Reporter
1	M	Right femoral pain due to pathological fracture of femoral diaphysis	3	7 years	None	None	Bilateral proximal femurs, right proximal tibia	Polyostotic	Nixon
2	F	Left hip discomfort and deformity of the left lower extremity	12	Unknown	None	None	Left proximal femur, left proximal tibia	Polyostotic	Nixon
3	M	Difficulty in running	5	13 years	Neck, buttock	None	Left proximal humerus	Polyostotic	Harris
4	M	Limp	4	30 years	Forehead, buttock, finger	None	Right distal humerus	Polyostotic	Harris
5	M	Left knee pain	13	8 months	None	None	Left proximal tibia	Monostotic	Takechi
The current case	F	Right knee pain	13	3 years	None	None	Right distal femur, right proximal tibia, right distal tibia	Polyostotic	
